# Symptomatic lumbosacral transitional vertebra: a review of the current literature and clinical outcomes following steroid injection or surgical intervention

**DOI:** 10.1051/sicotj/2017055

**Published:** 2017-12-12

**Authors:** Emil Kongsted Holm, Cody Bünger, Casper Bindzus Foldager

**Affiliations:** Orthopaedic Research Laboratory, Aarhus University Hospital, Aarhus Denmark

**Keywords:** Bertolotti’s, syndrome, Lumbosacral transitional vertebra, Steroid injection, Laminectomy, Spinal fusion

## Abstract

Bertolotti’s syndrome (BS) refers to the possible association between the congenital malformation lumbosacral transitional vertebra (LSTV), and low back pain (LBP). Several treatments have been proposed including steroid injections, resections of the LSTV, laminectomy, and lumbar spinal fusion. The aim of this review was to compare the clinical outcomes in previous trials and case reports for these treatments in patients with LBP and LSTV. A PubMed search was conducted. We included English studies of patients diagnosed with LSTV treated with steroid injection, laminectomy, spinal fusion or resection of the transitional articulation. Of 272 articles reviewed 20 articles met the inclusion criteria. Their level of evidence were graded I–V and the clinical outcomes were evaluated. Only 1 study had high evidence level (II). The remainders were case series (level IV). Only 5 studies used validated clinical outcome measures. A total of 79 patients were reported: 31 received treatment with steroid injections, 33 were treated with surgical resection of the LSTV, 8 received lumbar spinal fusion, and 7 cases were treated with laminectomy. Surgical management seems to improve the patient’s symptoms, especially patients diagnosed with “far out syndrome” treated with laminectomy. Clinical outcomes were more heterogenetic for patient’s treated with steroid injections. The literature regarding BS is sparse and generally with low evidence. Non-surgical management (e.g., steroid injections) and surgical intervention could not directly be compared due to lack of standardization in clinical outcome. Generally, surgical management seems to improve patient’s clinical outcome over time, whereas steroid injection only improves the patient’s symptoms temporarily. Further studies with larger sample size and higher evidence are warranted for the clinical guidance in the treatment of BS.

## Introduction

Bertolotti’s syndrome (BS) refers to the association of a lumbosacral transitional vertebra (LSTV) and low back pain (LBP) [1–6]. The LSTV is classified in different types and anatomic positions [2] (see Table 1) for further specifications.

**Table 1:** Definitions.

Hemisacralization	The transverse process of L5 forms a diarhrodial joint or a bony union with the sacrum unilaterally
Sacralization	The transverse process of L5 forms a diarhrodial joint or a bony union with the sacrum bilaterally
LSTV	Sacralization of the lowest lumbar vertebra and lumbarisation of the uppermost sacral segment. The LSTV can also form a diarthrodial joint or bony union the os ilium
LSTV articulation	Diarthrodial joint/pseudoarticulation/neoarthrosis between the transverse process of L5 and sacrum/os ilium
Bertolotti’s syndrome	LSTV association with low back pain (LBP) and radicular symptoms

The prevalence of an LSTV in the general population varies widely throughout the literature because of different diagnostic modalities and definitions, and hence the association of LSTV and LBP remains controversial. However, from a biomechanical and symptomatic standpoint it is important to distinguish the unilateral LSTV from the bilateral LSTV. According to a newly published review, the prevalence is estimated to be 4.0–35.9% with a mean of 12.3% [7]. The incidence of an LSTV in patients with LBP has been reported to be between 4.6% and 7% [4,8,9] and up to 11.4% in patients under the age of 30 years [4]. However, some authors did not find any association with the LSTV and lower back pain [8,9]. Therefore the clinical management of BS has been controversial and several treatments have been proposed for this syndrome, including local administered steroid injection and different surgical approaches [7].

The aim of the present review is to clarify the outcome for patients with BS treated with local steroid injection or with surgical intervention. We hypothesized that the clinical outcome would improve significantly for patients receiving surgical treatment versus steroid injections.

## Material and methods

**Figure 1:**
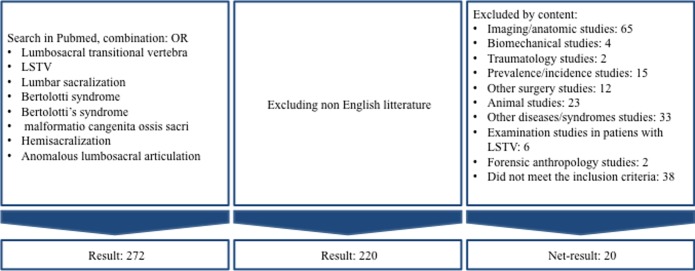
Inclusion flowchart.

We conducted a search in PubMed to evaluate the steroid and surgical treatment of BS. The search terms were “lumbosacral transitional vertebra” OR “LSTV” OR “lumbar sacralization” OR “Bertolotti syndrome” OR “Bertolotti’s syndrome” OR “malformation congenita ossis sacri” OR “hemisacralization” OR “anomalous lumbosacral articulation” (Figure 1). The search was performed on February 1, 2016.

Inclusion criteria were patients diagnosed with LSTV treated with: steroid injections, laminectomy, spinal fusion, or resection of the transitional articulation. Only English literature was included. From a total of 272 studies, 52 non-English articles were excluded. Of the remaining articles, we excluded an additional 200 articles by the content (Figure 1). Twenty articles met our inclusion criteria.

The articles were reviewed and the level of evidence graded in accordance to “Introducing levels of evidence to the journal” [10]. Clinical outcome were identified when present.

## Results

The identified papers included a total of 79 patients receiving steroid or surgical treatment for BS, which included 9 patients treated for the “far out foraminal” and “extraforaminal stenosis”. Of the 79 patients, 31 received treatment with steroid injections, 33 were treated with resection of the LSTV, 8 patients received lumbar spinal fusion and 7 patients were treated with laminectomy.

Twenty patients were diagnosed with a bilateral LSTV [11–20]:

– 10 patients treated bilaterally
  • 8 patients received steroid injections [12,13,20]
  • 1 patient treated with resection of the LSTV on both sides [16]
  • 1 patient surgically treated with lateral posterior fusion [18]
– 10 patients treated unilaterally
  • 5 reported only unilateral LBP [13,15,17]
  • 4 showed unilateral bony spurs [11,18,19]
  • 1 accepted only treatment in the most symptomatic side of LBP [16].

The remaining 54 patients were diagnosed with a unilateral LSTV (Tables 2–4). In five cases it is unclear whether the LSTV was uni- or bilateral [20,21]. We did not find any articles comparing surgical treatment and steroid injections for the treatment of BS. One study was a case–control study investigating LSTV resection and lumbar spinal fusion (level II). Four studies were cohorts with no controls (level IV). Fifteen papers were case-reports (level IV). There was no consensus in the use of outcome measures. Six articles used validated clinical outcome scores: visual analogue scale (VAS), numerical rating scale (NRS), Oswestry Disability Index (ODI), the Japanese Orthopaedic Association score (JOA), and/or Roland–Morris disability questionnaire [11,12,14,17,22,23]. The follow-up period, when reported, varied from 1 month to 12 years. Fourteen articles contained no specific validated measurements of clinical outcome, but described the patient’s outcome in subjective terms. In some of the studies it was not possible to identify when or if follow-up took place.

**Table 2:** Steroid injection.

Author	Pts	Treatment	Outcome	LSTV	F/U (mth)	LOE
Ichihara et al. [11]	1	^+^Two selective nerve root blocks	• Pain relief	Bilat.	12	IV
Mitra et al. [12]	1	Injection of steroid and Lidocaine	• ODI 33 → 26	Bilat.	1	IV
			• NRS 3/10 → 1/10			
Marks et al. [13]	10	X-ray guided lidocaine and steroid injection	• Immediately: 8 pt pain free	4 bilat.	41	IV
			• 1 w: 1 pt pain free	6 unilat.		
			• 1 d–12 w: 5 pts pain relapse			
			• 7–41 mth: 3 pts partial pain relief			
			• 2 yrs: 1 pt pain free			
Paraskewas et al. [25]	1	Local anaesthetic and steroid injection	• Improvement of symptoms	Unilat.	18	IV
Weber et al. [24]	1	^+^Two selective nerve root blocks	• Radiculopathy (only symptom) disappeared for 2 months	Unilat.	n/a	IV
Avimadje et al. [20]	12	11 pt received steroid injection. One pt refused treatment	• First hours: Improvement in pain	7 bilat.	24	IV
			• 1 mth: 9 pts 50% pain reduction	5 unilat.		
			• 6–24 mth: 7 of 8 pts symptom free			
Jain et al. [21]	4	Steroid injection	• 2 pts lost to follow-up	n/a	6	IV
Rodriquez et al. [26]	1	CT-guided steroid and anaesthetic injections	• Pain relief	Unilat.	n/a	IV

Pts: Number of patients, +: Treated for far out foraminal stenosis, ×: Patients, F/U: Follow-up, LOE: Level of evidence.

**Table 3:** LSTV resection.

Author	Pts	Outcome	LSTV	F/U (mth)	LOE
Jönsson et al. [27]	11	• 7 pts pain free	Unilat.	6–42	IV
		• 2 pts significant improvement in pain			
		• 2 pts no change in pain			
Santavirta et al. [14]	8	• Average disability (Oswestry scale): 30% (0–62%)	Unilat.	48–204	II
		• 6 pts with preoperative sciatic pain. Remained in 5 pts postoperative			
		• 5 pts with improvement of LBP (3 pain free)			
		• 3 pts reoperated (disc surgery, resection, anterior fusion)			
Brault et al. [23]	1	• Weeks: 90% relief of LBP and remission of leg pain	Unilat.	12	IV
		• 1 yr: No limitations in daily life activity and pain free	Unilat.	12	IV
Ugokwe et al. [29]	1	• 6 w: 10% improvement in pain	Unilat.	6	IV
		• 6 mths: 90% relief of LBP and lower extremity pain			
Almeida et al. [15]	2	• 6 and 12 mths: 1 pt pain free	1 unilat.	12	IV
			1 bilat.		
Malham et al. [28]	2	Patient 1:	Unilat.	24	IV
		• 4 w: Improvement in LBP and return to work			
		• 2 yrs: Working and performing low impact exercise			
		Patient 2:			
		• 3 mths: Improvement in LBP and returned to part time light work			
		• 2 yrs: Moderate work and performing low impact exercise			
Li et al. [16]	7	• 3 pts: Complete relief in LBP	5 unilat.	6–65	IV
		• 2 pts: Improvement in LBP	2 bilat.		
		• 3 pts: Complete relief of radicular pain			
		• 1 pt: Improvement of radicular pain			
Takata et al. [17]	1	• LBP: VAS: 80/100 → 29/100	Bilat.	n/a	IV
		• Sciatic pain: VAS: 80/100 → 10/100			

Pts: Number of patients, F/U: follow-up, LOE: level of evidence.

**Table 4:** Surgical nerve decompression.

Author	#	Approach	Outcome	LSTV	F/U (mth)	LOE
Abe et al. [18]	1	Anterior	• Immediately: Relive of LBP and leg pain	Bilat.	12	IV
			• “Several months”: Hypesthesia and numbness disappeared			
			• 1 yr: Returned to job. No LBP or numbness			
Ichihara et al. [11]	1	Dorsal	• Immediately: Hip and leg pain reduced	Unilat.	3	IV
			• 3 mths: No pain or numbness in the hip and leg			
			• JOA score: 14/29 → 22/29 (3 months postoperatively)			
			• JOA score 25/29 (2 years postoperatively)			
Weber et al. [24]	1	Dorsal	• 4 d: No leg pain	Unilat.	12	IV
			• 1 yr: No LBP or radicular pain			
Shibayama et al. [22]	1^×^	Dorsal	• Immediately: Relieved LBP and sciatic pain	Unilat.	30	IV
			• 30 mths: Walked well and returned to job			
			• VAS: 88/100 → 10/100			
			• JOA score: 10/29 → 25/29			
Miyoshi et al. [19]	1	Dorsal	• Immediately: Buttock and leg pain reduced	Bilat.	12	IV
			• 1 mth: Pain free			
			• 1 yr: Symptom free			
Kikuchi et al. [31]	2	Dorsal	Patient 1:	Unilat.	12	IV
			• Immediatly: LBP and leg pain disappeared			
			Patient 2:			
			• 6 mths: Leg pain decreased and complete recovery from muscle weakness			

#: Number of patients, ×: treated for extra foraminal stenosis, F/U: follow-up, LOE: level of evidence.

### Steroid injection

Eight studies were included. Only two studies investigated the clinical outcome of steroid injection in more than 4 patients [13,20]. The remainder were case-reports or data extracted from a study investigating different treatments of BS [21] (Table 2). The level of evidence was a level IV in all studies.

Marks et al. prospectively followed a cohort of 10 patients with severe LBP and diagnosed with an LSTV on X-ray [13]. They received X-ray-guided injections of steroids and local anesthetics in the LSTV. Eight patients had immediate total relief of pain and 1 patient had total pain relief within the first week. Five of those relapsed to their former pain level after 1 day to 12 weeks. Three patients reported adequate partial relief of pain after periods of 7 to 41 months and 1 patient remained pain free 2 years after the intervention.

In a retrospective study by Avimadje et al., 12 patients with LSTV reported same-side LBP or buttock-pain [20]. Eleven patients received steroid injection in the LSTV and 9 patients reported a 50% decrease in pain at 1-month follow-up. One patient refused treatment. Seven of 8 patients improved or had no symptoms 6–24 months later, two of which received a second injection of steroid one and two months, respectively, after the first injection.

Jain et al. prospectively reported 20 patients with BS describing different origins of pain and treatment methods of which, two patients were treated with steroid injections after a diagnostic block in the LSTV was preformed [21]. One patient had pain relief lasting 1 month, the other had pain relief lasting 3 months. None of the patients experienced pain relief at the end of the 6-month study period.

The remainders of the studies describing treatment with steroid injections [11,12,24–26] were case-reports [12,25,26] or studies, where the patients refused surgery after selective nerve root block [11,24]. Unfortunately, the follow-up period was not always reported (Table 2). Two cases have been reported on patients with bony spur from the LSTV articulation in the exit-zone of the root foramen causing impingement of the L5 nerve root [11,24]. Both received a selective nerve root block with steroid and local anesthetics, which caused immediate pain relief. The first case had no radiculopathy for two months and a repeat nerve root block was performed [24]. The study does not mention any subsequent clinical outcome. The second case did not have any pain at the 1-year follow-up. The JOA score was 7/29 before steroid injection but the JOA score at the follow-up was not reported.

### LSTV resection

We identified 8 studies addressing LSTV resection. Only 3 studies had a sample size of 4 patients or more (Table 3). One study was a level II prospective case–control study while the remainder were level IV evidence [14].

Jönsson et al., conducted a prospective study on 11 patients with persisting LBP, all treated with LSTV resection [27]. At follow-up (6–42 months, mean: 17 months) 7 patients experienced total pain alleviation, and additionally 2 patients experienced significant improvement. Two patients did not experience any changes in symptoms.

A prospective case–control study, by Santavirta et al., reported on 16 patients: 8 were treated with posterolateral spinal fusion, and were 8 treated with resection of the LSTV [14]. The control group received conservative treatment, not further specified. For the group treated with resection of the LSTV, five patients showed improvement of LBP, including 3 patients who were symptom free. ODI ranged from 8 to 62% (mean: 33.5%). Five of 6 patients with preoperative sciatic pain had persistent sciatic pain postoperative. One patient experienced onset of sciatic pain postoperative. Follow-up was 4–17 years; mean of 9 years.

Li et al., conducted a retrospective study including 7 patients with LBP, in which 6 also had radicular pain [16]. All were treated with resection of the LSTV, followed by a period of 6–65 months (mean: 21.6 months). Three patients experienced a total relief of LBP and radicular pain, and additionally 2 patients experienced permanent improvement of their LBP, and one of them also experienced improvement of the radicular pain. Two patients experienced initially improvement of LBP and radicular pain, but returned to former symptoms after 1 and 4 years respectively. One of them showed evidence of bone regrowth and underwent reoperation, which is not described any further in the article.

The last five studies were all case-reports [15,17,23,28,29]. Only one study used validated clinical outcome scores, and follow-up period was only reported in only four of them. For further specifications, see Table 3.

### Lumbar spinal fusion

Santavirta et al., presented the only study describing treatment of BS with lumbar spinal fusion in their prospective case–control study [14]. Five of eight patients in the lumbar spinal fusion group showed improvement of the LBP, including 4 showing no pain at all postoperatively. One patient did not show any improvement, and 2 patients showed increased pain. Seven patients reported preoperative sciatic pain and three of the patients had no sciatic pain postoperative, 4 continued with sciatic pain, and 1 patient reported onset of sciatic pain postoperative. ODI ranged from 0 to 48% (mean: 41.8%). The posterolateral spinal fusion group and resection of the LSTV group had comparable improvement in Oswestry score for pain. When the surgically treated group in total was compared with the conservatively treated matched control group, the surgical group had significantly better improvement. ODI was comparable between the groups. Adjacent level disc degeneration above the fused or resected level at follow-up (average 9 years) were found in 7 of 8 patients treated with posterolateral spinal fusion and 5 of 8 patients in the LSTV resection group.

### Surgical nerve decompression

We identified 6 studies representing 7 patients in the literature. Six of them with a bony spur from the LSTV leading to impingement of the nerve root at the foraminal exit zone and one patient presented an extraforaminal entrapment. LBP and sciatic pain was presented in all cases with a reported decrease in pain postoperatively. Two studies presented validated measurements for clinical outcome [11,22]. The follow-up period was in a range of 3–30 months and the level of evidence was IV in all studies. See Table 4 for further specifications.

## Discussion

The association between an LSTV and LBP is still controversial despite a high prevalence [7]. The literature regarding the local administered steroid injection and surgical management is very sparse with only 79 identified cases in this present review. A comparative analysis in this review is challenging due to the small number of cases, absence of defined evaluation criteria, lack of control groups (except Santavirta et al. [14]), and shortage of standardization in data collection. The level of evidence in reports on treatment of BS is very low with only 1 article being level II and the remainder level IV. The different studies did not distinguish between uni- and bilateral LSTV and types of LSTV in their results. A diagnostic block was described in 8 studies leading to the uncertainty of whether the pain generator was the LTSV or the stressed level above [12,13,16,17,20,25,26,28].

This leads to a delay in the diagnostic and clinical management of BS, which is a potential differential diagnosis in patients with LBP, especially in patients under the age of 30 where BS has been reported as high as 11.4% [4].

There is a paucity of studies regarding the biomechanical effects in the presence of an LSTV. To our knowledge, no studies have investigated the potential biomechanical consequences of unilateral LSTV versus bilateral. The included articles did not distinguish between the different presentations in their results for clinical outcome.

It has been proposed, that disturbance of biomechanics in the lowest segment of the lumbar spine, caused by the unilateral anomalous articulation, could be a pain generator [27]. Furthermore, it has been suggested that the biomechanical stress transferred to the upper mobile vertebral segment, can accelerate early disc degeneration at the adjacent levels, leading to disc protrusion or extrusion, which can cause LBP and sciatic pain [8].

Other studies have shown an increased risk of disc degeneration in the lumbar discs immediately above the transitional vertebra [2,3,30]. This may be due to a weak iliolumbar ligament above the transitional vertebra [3]. Identical findings were seen in the case–control study from Santavirta et al., where 5 out of 8 patients treated with resection of the LSTV had degenerative changes at the adjacent segment above at follow-up [14]. Some authors argue that these findings could influence the choice of treatment towards an instrumentation and fusion of the lumbar back instead of excision of the pseudo-articulation between the transverse process of L5 and sacrum [28]. In contrast Santavirta et al. found degenerative changes in the first disc above the fused segments in 7 out of 8 patients at follow-up [14].

The Castellvi classification of the LSTV, ranging from enlargement of the transverse process of L5 to complete fusion with the sacral bone, is used in 8 of the 20 included articles [11,18,19,21,22,24,25,31]. An LSTV articulation or fusion to the iliac bone was found in 4 articles [12,14,20,27]. The 60 cases, from which the classification is based could represent a limitation in the Castellvi classification [2]. To our knowledge, no other studies have included the iliac bone in the LSTV classification.

In 1984, Wiltse et al. introduced “Far out Syndrome” as an entrapment of the L5 nerve root between the transverse process of L5 vertebra and the sacral ala seen in elderly patients with degenerative lumbar scoliosis and younger patients with isthmic spondylolisthesis [32]. The entrapment of the L5 nerve root is similar in the presented cases treated with laminectomy, but the source of pain is different here, with a bony spur from the transverse process of L5 in the LSTV causing the foraminal and extraforaminal compression. In 7 cases the entrapment occurred at the exit zone of the foramina, but Shibayama et al. presented a case, where the entrapment point was in the extraforaminal zone [22]. Neural compression caused by a bony spur has been reported with a prevalence of 13% in patients with an LSTV and can be symptomatic in up to 70% of these patients [33]. These case presentations of nerve root entrapment caused by bony spurs from the LSTV are in favor of more heterogenic pathological causes of LBP and sciatic pain in patients with BS. In some cases the pain might be due to the LSTV pseudo-articulation itself, or it could be due to a bony spur from the LSTV.

## Conclusion

Very few studies have investigated the treatment of BS. The clinical outcome measures are heterogenic and the level of evidence was generally low. No studies compared surgical treatment versus steroid injections for the treatment of BS and they did not distinguish between unilateral versus bilateral LSTV in their results. Therefore, best practice for treatment of BS cannot be determined by the present literature. Further studies with larger sample sizes and longer follow-up periods are warranted for the clinical guidance in the treatment of BS.

## Conflict of interest

EKH, CB and CBF have no conflicts of interest related to this work.
